# *Salmonella* Outbreaks Associated with Not Ready-to-Eat Breaded, Stuffed Chicken Products — United States, 1998–2022

**DOI:** 10.15585/mmwr.mm7218a2

**Published:** 2023-05-05

**Authors:** Laura Ford, Sean Buuck, Taylor Eisenstein, Andrea Cote, Zachary D. McCormic, Selena Kremer-Caldwell, Bonnie Kissler, Matthew Forstner, Alida Sorenson, Matthew E. Wise, Kirk Smith, Carlota Medus, Patricia M. Griffin, Misha Robyn

**Affiliations:** ^1^Division of Foodborne, Waterborne, and Environmental Diseases, National Center for Emerging Zoonotic and Infectious Diseases, CDC; ^2^Minnesota Department of Health; ^3^Food Safety and Inspection Service, U.S. Department of Agriculture, Washington, DC; ^4^Minnesota Department of Agriculture.

Not ready-to-eat (NRTE) breaded, stuffed chicken products (e.g., chicken stuffed with broccoli and cheese) typically have a crispy, browned exterior that can make them appear cooked. These products have been repeatedly linked to U.S. salmonellosis outbreaks, despite changes to packaging initiated in 2006 to identify the products as raw and warn against preparing them in a microwave oven (microwave) (*1*–*4*). On April 28, 2023, the U.S. Department of Agriculture proposed to declare *Salmonella* an adulterant* at levels of one colony forming unit per gram or higher in these products (*5*). *Salmonella* outbreaks associated with NRTE breaded, stuffed chicken products during 1998–2022 were summarized using reports in CDC’s Foodborne Disease Outbreak Surveillance System (FDOSS), outbreak questionnaires, web postings, and data from the Minnesota Department of Health (MDH)^†^ and the U.S. Department of Agriculture’s Food Safety and Inspection Service (FSIS). Eleven outbreaks were identified in FDOSS. Among cultured samples from products obtained from patients’ homes and from retail stores during 10 outbreaks, a median of 57% of cultures per outbreak yielded *Salmonella*. The NRTE breaded, stuffed chicken products were produced in at least three establishments.^§^ In the seven most recent outbreaks, 0%–75% of ill respondents reported cooking the product in a microwave and reported that they thought the product was sold fully cooked or did not know whether it was sold raw or fully cooked. Outbreaks associated with these products have occurred despite changes to product labels that better inform consumers that the products are raw and provide instructions on safe preparation, indicating that consumer-targeted interventions are not sufficient. Additional *Salmonella* controls at the manufacturer level to reduce contamination in ingredients might reduce illnesses attributable to NRTE breaded, stuffed chicken products.

State health officials submit reports of foodborne *Salmonella* outbreaks to FDOSS. A foodborne outbreak is defined as the occurrence of a similar illness associated with a common food exposure in two or more persons. *Salmonella* outbreaks with mention of frozen, breaded, or stuffed chicken that occurred during 1998–2022 were identified in FDOSS; data for 2022 are incomplete. Search results were supplemented by information obtained from outbreak investigators, and a review of questionnaires administered to ill persons, web postings, product recall information, product sampling results, and data from MDH and FSIS.

Descriptive analyses were performed using R statistical software (version 4.0.2; R Foundation). The number of outbreaks, illnesses,^¶^ and hospitalizations; serotypes; setting where food was prepared; patient sex and age groups; type of chicken used in the products; testing results for food and environmental samples collected from patients’ homes or retail establishments; and product recalls were summarized. Product type and producing establishments were identified through traceback by Minnesota’s Departments of Agriculture and Health or by FSIS.

To examine the cooking practices of ill persons in outbreaks, and whether they believed the product was sold fully cooked, data collected from patient interviews during Minnesota and multistate outbreak investigations were summarized. Responses from outbreaks occurring during 2008–2021, after labeling changes were initiated in 2006, were compared with those from earlier outbreaks. This activity was reviewed by CDC and was conducted consistent with applicable federal law and CDC policy.**

An NRTE breaded, stuffed chicken product was the confirmed^††^ food vehicle in 11 *Salmonella* outbreaks^§§^ that occurred during 1998–2022, comprising 187 cases of illness, 42 hospitalizations, and no deaths (Table 1). The outbreaks were caused by *Salmonella* serotypes Enteritidis (seven outbreaks), Typhimurium (two), Heidelberg (one), and I 4,[5],12:i- (one). In 10 of the 11 outbreaks, the products were prepared in private homes. Outbreak illnesses were identified in 21 U.S. states; five of six single-state outbreaks occurred in Minnesota and illnesses occurred in Minnesota in every multistate outbreak. Among 139 patients for whom data from 10 outbreaks were available, 73 (53%) were male and 64 (42%) of 153 patients were aged 20–49 years.

**TABLE 1:** *Salmonella* outbreaks associated with not ready-to-eat breaded, stuffed raw chicken products — United States, 1998–2022*

Year of first illness	State where exposure occurred (no. of states)^†^	*Salmonella* serotype	No. of patients	No. of hospitalizations	Site of food preparation	Implicated establishment	Product recall^§^
1998	Minnesota	Typhimurium	33	0	Private home	A	Yes
2005	Multistate (2)	Heidelberg	9	4	Private home	B	No^¶^
2005	Multistate (10)	Enteritidis	41	6	Private home	A, B, and C	Yes^¶^
2006	Minnesota	Typhimurium	3	2	Private home	B	No
2008	Minnesota	Enteritidis	7	3	Private home	A	No^¶^
2008	Multistate (2)	I 4,[5],12:i-	19	8	Private home and workplace cafeteria	A and C**	No^¶^
2013	Washington	Enteritidis	10	0	Camp	Unknown	No
2014	Minnesota	Enteritidis	8	1	Private home	B	Yes
2015	Minnesota	Enteritidis	6	2	Private home	B	Yes^¶^
2015	Multistate (7)	Enteritidis	15	4	Private home	C	Yes^¶^
2021	Multistate (11)	Enteritidis	36	12	Private home	A	Yes^¶^

**Abbreviations:** FDOSS = Foodborne Disease Outbreak Surveillance System; FSIS = Food Safety and Inspection Service; USDA = U.S. Department of Agriculture.

* Minnesota Department of Health and FSIS investigated four additional clusters of illness that were suspected to be associated with not ready-to-eat breaded, stuffed chicken products; none met the National Outbreak Reporting System definition of an outbreak (https://www.cdc.gov/nors/downloads/guidance.pdf). One FDOSS-identified outbreak was a single state outbreak without notification to FSIS. No outbreaks were detected during 2022.

^†^ 2005 multistate Heidelberg: Minnesota and Michigan; 2005 multistate Enteritidis: California, Colorado, Illinois, Iowa, Maryland, Minnesota, North Dakota, Oklahoma, Pennsylvania, and Tennessee; 2008 multistate: Minnesota and Wisconsin; 2015 multistate: Connecticut, Illinois, Minnesota, New Hampshire, New York, Oklahoma, and Wisconsin; 2021 multistate: Arizona, Arkansas, Connecticut, Illinois, Indiana, Michigan, Minnesota, Nevada, New York, Ohio, and Oklahoma.

^§^ Product recall was considered nationwide for outbreaks during 2005–2018. It is unknown whether the 1998 recall was nationwide. USDA FSIS determines a recall to be nationwide when more than 11 states are involved in the recall.

^¶^ Public health alert issued by FSIS. No recall from commerce by the company in the 2005 multistate outbreak, but one grocery store chain voluntarily removed all product from shelves.

** Additional brands of frozen stuffed chicken products were mentioned by patients in one outbreak but not traced back; therefore, the producers were unknown.

NRTE breaded, stuffed chicken products were made with comminuted^¶¶^ chicken in the three 2015 and 2021 outbreaks. The type of chicken used during earlier outbreaks was not documented. FSIS issued product recalls in six outbreaks and public health alerts in seven.*** Product samples were collected from patients’ homes in nine outbreaks and from retail stores in 10.^†††^ A median of 57% (range = 0%–100%) of samples per outbreak from patients’ homes and 57% (range = 11%–93%) of samples per outbreak from retail stores yielded *Salmonella* upon culture. An isolate from at least one product matched the patients’ serotype or strain in every outbreak in which product was sampled. The producing establishment was unknown for one outbreak; products in the other 10 outbreaks came from at least three producing establishments. Establishments A and B were implicated in five outbreaks each, and establishment C was implicated in three outbreaks. Establishments A and C are still operating; they are two of the six U.S. establishments that make these products.

Among 47 patients in four outbreaks during 1998–2006 (before product labeling changes) who provided data on the appliance used to cook the product, a median of 85% per outbreak (range = 67%–100%) reported cooking the product in a microwave; among 57 patients who provided these data from seven outbreaks during 2008–2021 (after labeling changes), a median of 20% per outbreak (range = 0%–75%) reported cooking the product in a microwave (Table 2). In four outbreaks that occurred during 1998–2006, among 37 patients who provided information about whether they thought the product was sold fully cooked, a median of 56% per outbreak (range = 33%–100%) reported that they thought the product was sold fully cooked or did not know whether it was sold raw or fully cooked. In six outbreaks during 2008–2021, among 34 patients who provided this information, a median of 27% per outbreak (range = 0%–75%) thought the product was sold fully cooked or did not know whether it was sold raw or fully cooked.

**TABLE 2:** Cooking appliances used by ill persons affected by not ready-to-eat breaded, stuffed raw chicken *Salmonella* outbreaks, by year and serotype — United States, 1998–2022*

Year	State	Serotype	No. of ill persons who responded/No. asked (%)			
			Appliance used to cook product			Thought or didn’t know if product was sold fully cooked
			Microwave oven	Conventional oven	Other	
1998	Minnesota	Typhimurium	10/15 (67)	5/15 (33)	0/15 (—)	10/16 (63)
2005	Multistate	Heidelberg	2/2 (100)	0/2 (—)	0/2 (—)	2/2 (100)
2005	Multistate	Enteritidis	19/27 (70)	7/27 (26)	1/27 (4); toaster oven	8/16 (50)
2006	Minnesota	Typhimurium	3/3 (100)	0/3 (—)	0/3 (—)	1/3 (33)
2008	Minnesota	Enteritidis	5/7 (71)	2/7 (29)	0/7 (—)	3/4 (75)
2008	Multistate	I 4,[5],12:i-	9/12 (75)	3/12 (25)	0/12 (—)	2/6 (33)
2013	Washington	Enteritidis	0/10 (—)	0/10 (—)	10/10 (100); stovetop	Unknown
2014	Minnesota	Enteritidis	1/5 (20)	4/5 (80)	0/5 (—)	0/3 (—)
2015	Minnesota	Enteritidis	1/5 (20)	3/5 (60)	1/5 (20); convection-microwave combination oven	1/5 (20)
2015	Multistate	Enteritidis	0/5 (—)	4/5 (80)	1/5 (20); toaster oven	1/5 (20)
2021	Multistate	Enteritidis	5/13 (38)	6/13 (46)	2/13 (15); air fryer	5/11 (45)

* No outbreaks were detected in 2022.

## Discussion

Eleven *Salmonella* outbreaks linked to NRTE breaded, stuffed chicken products (involving 187 patients) were reported in the United States during 1998–2022. Most of the products tested contained *Salmonella*. Products were produced by at least three establishments. Outbreaks have continued to occur despite changes made to product labels to better inform consumers and increase the percentages of persons who understand that the product is sold raw. Thus, stronger controls are needed to prevent illnesses associated with these products.

NRTE breaded, stuffed chicken products can be made with various types of chicken, including comminuted, trimmings, or other parts. Certain chicken types are subject to FSIS performance standards, which are used to measure an establishment’s process control; the comminuted chicken used to make these products has the highest allowable percentage (13 of 52 [25%]) of *Salmonella* positives (*6*). On April 28, 2023, the U.S. Department of Agriculture proposed to declare *Salmonella* an adulterant in NRTE breaded, stuffed chicken products, meaning that the product will be subject to regulatory action if *Salmonella* is detected even at very low levels (*5*).

Canada enacted regulations for certain breaded chicken products after investigators identified 19 *Salmonella* outbreaks caused by NRTE breaded chicken products during 2015–2019 (*7*). These products were not stuffed; most were chicken nuggets. The government introduced four control options in nonstuffed products to reduce *Salmonella* to below detectable amounts in these products (*7*). In 2019, the incidence of illness caused by *Salmonella* Enteritidis, the serotype implicated in 89% of those outbreaks, was 33% lower than it was during 2015–2018 and 7% lower than during the baseline years 2010–2014 (*8*).

Consumer-based interventions alone, such as improved product labels, have not been an effective solution. In recent years, labels have recommended using a conventional oven rather than a microwave and using a food thermometer (*3*); however, a consumer research study found that even when consumers read the label, 12% did not realize the product was raw or partially cooked, and among consumers who owned a food thermometer, 52% reported that they typically did not use it while preparing this product (*9*). Although labeling changes can help protect consumers, the questionnaire data show that some persons who knew the product was raw and followed the cooking instructions still became ill. Moreover, label changes cannot address inequities in access to recommended cooking appliances (*3*).

The number of patients who became ill from these products is likely much higher than that indicated from outbreak reports. Many persons regularly eat these products: in a U.S. population survey, 7.4% reported eating these products in the previous week.^§§§^ Although implicated products were distributed nationally, MDH officials identified all multistate outbreaks, and almost one half of outbreaks had cases identified only in Minnesota, suggesting that some outbreaks occurred but were not identified in other states.

Illnesses even among persons who reported that they used a conventional oven and knew the product was raw indicate that consumer-based interventions have been insufficient. The high rate of contamination of products in outbreaks and the lack of first recognition of multistate outbreaks by a state other than Minnesota suggest that the prevalence of illness due to these products is higher than that indicated by outbreaks. Moreover, only a small proportion of all *Salmonella* illnesses are identified as such. Illness could be reduced by enhanced *Salmonella* control at the manufacturers that produce these products. The U.S. Department of Agriculture’s proposal to declare *Salmonella* an adulterant in NRTE breaded and stuffed chicken products will bring additional focus to this public health problem and encourage producers to better control *Salmonella* in the ingredients used to produce these products.

**Summary**

**What is already known about this topic?**

Not ready-to-eat breaded, stuffed chicken products have repeatedly been a source of *Salmonella* outbreaks. On April 28, 2023, the U.S. Department of Agriculture proposed to declare *Salmonella* an adulterant in these products.

**What is added by this report?**

During 1998–2022, 11 *Salmonella* outbreaks linked to these products were reported; 57% of samples per outbreak from patient homes and retail stores yielded *Salmonella*. Outbreaks continue to occur, although a smaller percentage of patients reported cooking the product in a microwave after labeling changes.

**What are the implications for public health practice?**

Outbreaks have continued despite consumer-based interventions. Additional control measures for *Salmonella* contamination by manufacturers could reduce *Salmonella*-involved illnesses associated with these products.

## Footnotes

* The circumstances under which a poultry product is considered adulterated are defined in the Poultry Products Inspection Act, which includes several conditions that result in a product’s being unsafe or harmful to health (21 U.S.C. 453 [g]).

† With one exception, MDH first identified and led the investigation for every outbreak described in this report, and also provided CDC with additional outbreak reports for each of the 10 outbreaks.

§ An establishment at which inspection of the slaughter of poultry, or the processing of poultry products, is maintained under the authority of 21 U.S.C. Chapter 10 Sect. 453p, as determined by the U.S. Department of Agriculture secretary.

¶ Illnesses include both laboratory-confirmed and epidemiologically linked to a laboratory-confirmed illness or implicated food. https://www.cdc.gov/nors/downloads/guidance.pdf

** 45 C.F.R. part 46.102(l)(2), 21 C.F.R. part 56; 42 U.S.C. Sect. 241(d); 5 U.S.C. Sect. 552a; 44 U.S.C. Sect. 3501 et seq.

†† NRTE breaded, stuffed chicken products were considered the confirmed food vehicle when there was at least one type of evidence (epidemiologic, traceback, or laboratory testing) in point source outbreaks or at least two types of evidence in outbreaks with exposures in multiple venues or in more than one county or state. https://www.cdc.gov/nors/downloads/guidance.pdf

§§ MDH and FSIS investigated four additional clusters of illness suspected to be associated with NRTE breaded, stuffed chicken products; none met the National Outbreak Reporting System outbreak definition.

¶¶ Products that have been ground, mechanically separated, or hand- or mechanically deboned and further chopped, flaked, minced, or otherwise processed to reduce particle size.

*** FSIS defines a recall as a firm’s action to remove product from commerce to protect the public from consuming adulterated or misbranded product. Although recalling product is a firm’s decision, FSIS coordinates with the firm to ensure it has properly identified and removed recalled product from commerce by verifying the effectiveness of the firm’s recall activities. FSIS also notifies the public about product recalls. A public health alert is issued when there is reason to believe a meat or poultry product might be associated with human illness, but FSIS cannot identify a specific product that could be recalled (Directive 8080.1, 2013). Some of the outbreaks had both a public health alert and recall, some only a public health alert, and others only a recall.

††† No product samples were collected in the 2013 Washington *Salmonella* Enteritidis outbreak, and no product samples were taken from patients’ homes in the 2005 multistate *Salmonella* Heidelberg outbreak. Product sampling during the 2005 multistate *Salmonella* Enteritidis outbreak and the 2006 Minnesota *Salmonella* Typhimurium outbreak occurred together and the proportion of samples yielding *Salmonella* was only counted once in median calculations.

§§§
https://wwwn.cdc.gov/Foodnetfast/PopSurvey
